# Accelerating recovery from jet lag: prediction from a multi-oscillator model and its experimental confirmation in model animals

**DOI:** 10.1038/srep46702

**Published:** 2017-04-26

**Authors:** Hiroshi Kori, Yoshiaki Yamaguchi, Hitoshi Okamura

**Affiliations:** 1 Department of Information Sciences, Ochanomizu University, Tokyo, 112-8610, Japan; 2 Department of Systems Biology, Graduate School of Pharmaceutical Sciences, Kyoto University, Kyoto, 606-8501, Japan

## Abstract

The endogenous circadian clock drives oscillations that are completely synchronized with the environmental day–night rhythms with a period of approximately 24 hours. Temporal misalignment between one’s internal circadian clock and the external solar time often occurs in shift workers and long-distance travelers; such misalignments are accompanied by sleep disturbances and gastrointestinal distress. Repeated exposure to jet lag and rotating shift work increases the risk of lifestyle-related diseases, such as cardiovascular complaints and metabolic insufficiencies. However, the mechanism behind the disruption of one’s internal clock is not well understood. In this paper, we therefore present a new theoretical concept called “jet lag separatrix” to understand circadian clock disruption and slow recovery from jet lag based on the mathematical model describing the hierarchical structure of the circadian clock. To demonstrate the utility of our theoretical study, we applied it to predict that re-entrainment via a two-step jet lag in which a four-hour shift of the light-dark cycle is given in the span of two successive days requires fewer days than when given as a single eight-hour shift. We experimentally verified the feasibility of our theory in C57BL/6 strain mice, with results indicating that this pre-exposure of jet lag is indeed beneficial.

An abrupt change in one’s daily rhythm, which people often experience on shift work and long-distance trips, can cause health problems, including exhaustion, loss of concentration, and loss of appetite^1^,^2^,^3^. Moreover, repeated exposure to jet lag and shift work can increase the risk of diseases, including cancers, diabetes, and hypertension^4^,^5^,^6^,^7^,^8^. Therefore, it is important to understand the mechanism underlying such problems and, if possible, the means to reducing such problems by altering approaches to life management and work schedules.

Almost all the cells throughout the body exhibit circadian rhythm generated by the oscillation of the core clock genes. These cellular rhythms are orchestrated at the system level by the suprachiasmatic nucleus (SCN) in the hypothalamus. The SCN consists of thousands of clock cells, with each clock cell carrying different types of neurotransmitters and receptors^9^. These cells form a neural network that produces robust oscillations^10^. In short, the coherent output signal from the SCN cells synchronizes cellular clocks throughout the body^11^,^12^,^13^, which enables organisms to keep rhythmic activities, such as sleep-awake cycle, blood pressure, core body temperature, hormonal production, and other biological activities^11^,^12^,^13^.

Jet lag can be emulated in laboratory experiments by presenting an abrupt shift of the light-dark (LD) cycle to animals. Recently, in our experimental jet-lag paradigm, we reported that the oscillatory amplitude of clock genes in the SCN is substantially dampened just after advancing the LD cycles by eight hours^14^. Although synchronized rhythms of clock gene transcriptions were found across thousands of neurons within the mammalian SCN under normal conditions, our study of the SCN sections presented in ref. 14 suggests that cell-to-cell synchrony was considerably disrupted, which is likely to be the reason for the damped oscillation of clock gene expression. Moreover, in mice genetically lacking both V1a and V1b receptors (i.e., *V*1*a*^−/−^*V*1*b*^−/−^), which are receptors of an SCN-enriched neurotransmitter arginine vasopressin (AVP), the dampening of the oscillation of clock genes was only subtle after advancing the LD cycle. These results suggest that three concepts—i.e., strong intercellular coupling in the SCN, large desynchrony among SCN neurons just after advancing LD cycle, and slow adaptation to the new LD cycle—are closely interrelated.

A mathematical approach is essential for understanding complex multi-cellular dynamics and emergent properties. Although there is a large body of mathematical studies focused on the spontaneous synchronization of a population of clock cells^15^,^16^,^17^,^18^,^19^,^20^,^21^,^22^,^23^, only a few studies have addressed the response to substantial jet lag in multicellular systems^14^,^24^,^25^,^26^. In prior work, we proposed a mathematical model to investigate the role of intercellular coupling in the SCN during the adaptation process after substantial jet lag^14^. As shown in Fig. 1, our model consists of three oscillators with a structure based on the anatomical properties of the SCN. The SCN is composed of a heterogeneous population of neurons utilizing specific neurotransmitters. Oscillator 0 represents a group of neurons receiving direct input from the retina, which uses vasoactive intestinal peptide (VIP) as a neurotransmitter to send signals to the entire SCN. Oscillators 1 and 2 represent groups of neurons not receiving direct input from the retina; these neurons use multiple neurotransmitters, such as AVP and GABA, to interact with one another. Our model successfully reproduced our main experimental finding that the adaptation to the advanced LD cycle was accelerated when the intercellular coupling is weakened. However, its mechanism remains rather vague.

In this paper, we therefore propose a minimal model to elucidate the mechanism of desynchrony, its resultant effect on the adaptation process, and the role of intercellular coupling. Further, we shed light on a key theoretical concept referred to as “jet lag separatrix”, which turns out to be crucial for understanding desynchrony and slow adaptation under jet lag conditions. Our model is based on our previous model^14^,^25^, but is further simplified, thus being advantageous in terms of gaining a clear understanding. Moreover, we here propose a feasible method to accelerate the re-entrainment process for jet lag corresponding to a long-distance eastbound trip. We experimentally verified our method using mice, observing the re-entrainment process to be in good agreement with our mathematical predictions. We also discuss a relevant application of our study to shift work.

## Model

Our model consists of the following set of differential equations for SCN cells:


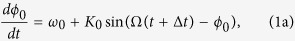



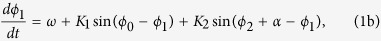



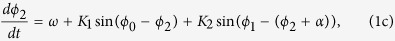


where *t* [day] is time, *ϕ*_*i*_(*t*) (0 ≤ *ϕ* < 2*π*) is the phase of oscillator *i (i* = 0, 1, 2); Ω = 2*π* is the frequency of the LD cycle (corresponding to the period of exactly one day); *ω*_0_ is the frequency of oscillator 0; and *ω* is the frequency of oscillators 1 and 2.

Jet lag, i.e., a phase shift of the LD cycle, is described as Δ*t* = 0 for *t* < *t*_jetlag_ and Δ*t* = *δ* for *t* > *t*_jetlag_, where *δ* is the jet lag and *t*_jetlag_ is the time at which jet lag occurs. In the context of long-distance trips, *t* and *t* + *δ* are the local times of the departure and destination places, respectively, with positive *δ* 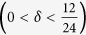 and negative *δ* 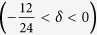 corresponding to eastbound and westbound trips, respectively.

The second term in equation (1a) describes the influence of the LD cycle on oscillator 0, where *K*_0_ is interpreted as the product of the light intensity and the sensitivity of oscillator 0 to light stimuli. For simplicity, we assume that *K*_0_ is so large that oscillator 0 is always instantaneously entrained to the LD cycle. The entrainment condition for oscillator 0 is 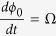, resulting in


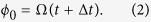


We thus use equation (2) instead of equation (1a). Note that *ω*_0_ value is irrelevant given the assumption of large *K*_0_.

Oscillators 1 and 2 are affected by oscillator 0 through VIP, as described by the second terms in equations (1b) and (1c) with strength *K*_1_. This VIP coupling enables our entire system to entrain to the LD cycle. It also makes oscillators 1 and 2 mutually synchronized even in the absence of other coupling terms. Moreover, oscillators 1 and 2 mutually interact with one another, as described by the third terms with strength *K*_2_. As we detail later, this interaction results in the synchronization of these two oscillators with some phase lag (i.e., out-of-phase synchrony). Parameters *ω* and *α* play important roles in the behavior of this clock system. As rationalized below, we will employ the following parameter values: *ω* = 2*π* − 0.19; *K*_1_ = 1.0; *K*_2_ = 1.4; and *α* = 2.0.

Note that every interaction function in our model is given as a function of the phase difference between two interacting oscillators, which is broadly used in the study of synchronization^27^,^28^,^29^,^30^,^31^. A more realistic choice here could be the product of the functions of phases^15^, e.g., *K*_1_*Z(ϕ*_1_)*P(ϕ*_0_) instead of the second term in equation (1b), where *Z(ϕ*_1_) and *P(ϕ*_0_) are the phase response function of oscillator 1 and a signal coming from oscillator 0, respectively. In our previous study^14^, we chose the latter type. As we show below, dynamical behavior in our present model is qualitatively the same as that of our previous study. Because we expect that our results do not qualitatively depend on the choice of interaction types and particular functional forms, we choose the present interaction form because of its mathematical tractability.

Continuing with our model, we assume that oscillators 1 and 2 are the primary contributors to the clock gene expression and circadian behavior since the dorsomedial part of the SCN where AVP is highly expressed is thought to be the source of the circadian output of the whole SCN^32^. Especially, the dorsomedial hypothalamic nucleus, a key region for behavioral circadian rhythm, is exclusively innervated by the afferents from the dorsomedial part of the SCN^33^,^34^. Considering the expression level of *i* is 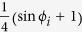, the total level of clock gene expression is given by


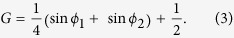


For convenience, we introduce synchronization level *R(t*) and mean phase Φ(*t*) of oscillators 1 and 2 as


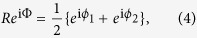


where i denotes 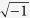. Here, *R* is often called the Kuramoto order parameter, which assumes 1 and 0 for in-phase and anti-phase synchronization, respectively. Further, Φ is essentially the same as (*ϕ*_1_ + *ϕ*_2_)/2 (see Supporting Information C), which we thus call the mean phase. More precisely, when two oscillators are located on the unit circle centered at the origin, the length between the origin and the center of mass of these oscillators is *R*; further, the angle of the center of mass of these oscillators seen from the origin is Φ. One can show that 
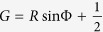
, thus the higher the synchronization level, the stronger the oscillation amplitude.

Our model reproduces the following important properties of the clock system in model animals: (i) phase dispersion; (ii) jet lag separatrix; (iii) desynchrony and slow re-entrainment for advance phase shifts. Each of these properties is described in further detail below.

### Phase dispersion

The phases of clock cells in the SCN are well coordinated but widely dispersed^10^. Such dispersion may result from the heterogeneity of the properties of individual cells or the nature of the interactions among cells. In our previous study, we found that after the phases of clock cells were reset by the administration of cycloheximide, the synchrony of clock cells in wild type (WT) mice recovered faster than that in *V*1*a*^−/−^*V*1*b*^−/−^ mice^14^; here, AVP is likely to contribute to synchronization. We therefore model the interaction between oscillators 1 and 2 as one that facilitates synchronization with a phase difference between oscillators, i.e., out-of-phase synchrony. Although we consider that this interaction is mediated by multiple neurotransmitters, for simplicity, we refer to it as the AVP interaction.

The interaction yielding out-of-phase synchrony can concisely be described by the third term in equations (1b) and (1c). Its effect can be clearly understood in the case of *K*_1_ = 0. In such a case, the synchronized state between oscillators 1 and 2 is obtained by solving 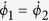 where 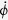 denotes 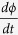, resulting in Δ*ϕ* = *α*, where Δ*ϕ* = *ϕ*_1_ − *ϕ*_2_. Thus, *α* controls the phase difference between oscillators 1 and 2. Because the VIP interaction in our model facilitates the in-phase synchronization between oscillators 1 and 2, the phase difference decreases as *K*_1_ increases. We can roughly estimate


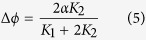


in the presence of both VIP and AVP interactions (see Supporting Information B).

### Jet lag separatrix

When an organism experiences a large phase shift in its environmental rhythm, there are two ways to adapt to the shift, i.e., either advancing or delaying one’s clock with respect to the local time. Therefore, we expect there to be critical jet lag *δ*_*c*_ at which the way to adapt switches, a quantity we refer to as the jet lag separatrix. Mathematically, this phenomenon is characterized by an unstable solution, which generally emerges when an oscillatory system is entrained by periodic stimuli^35^,^36^. In mice, we found that the jet lag separatrix is between the phase advances by eight hours and 12 hours of the LD cycle (see Supplementary Information A).

The main determinants of jet lag separatrix in our model are natural frequency *ω* and strength *K*_1_ of the VIP interaction. The jetlag separatrix can be obtained analytically in the case of *K*_2_ = 0. In this case, oscillators 1 and 2 are identical, and from equations (1b) and (2), relative phase *ψ*_*i*_ = *ϕ*_*i*_ − Ω*t (i* = 1, 2) obeys


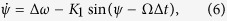


where Δ*ω* = *ω* − Ω and subscript *i* is dropped for better presentation. The entrained state is obtained by solving 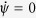. A pair of constant solutions exist when 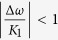. Before jet lag is introduced (*t* < *t*_jetlag_, Δ*t* = 0), the solutions are *ψ* = *ψ*_s_ (stable) and *ψ*_u_ (unstable), where 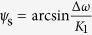 and 
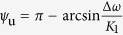
, as illustrated in Fig. 2(a). After jet lag occurs (*t* ≥ *t*_jetlag_, Δ*t* = *δ*), the constant solutions are rotated by Ω*δ*, i.e., 
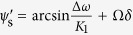
 and 
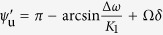
.

Next, we consider an adaptation process for *t* > *t*_jetlag_. At *t* = *t*_jetlag_, *ψ(t*) starts from former stable state *ψ* = *ψ*_s_, represented as gray circles in Fig. 2(b,c), and approaches new stable state 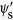 for *t* > *t*_jetlag_. Whether *ψ(t*) increases or decreases with time (i.e., the clock is advanced or delayed) depends on the relation between *ψ*_s_ and 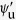. For example, suppose Δ*ω* < 0 and we advance the phase of the LD cycle (i.e., 0 < *δ* < 12/24). Before jet lag, the flow of *ψ(t*) is as shown in Fig. 2(a). For a small *δ* value, the clock adjusts by advancing its phase, because the flow direction around *ψ*_s_ is counterclockwise, as depicted in Fig. 2(b). Conversely, for a large *δ* value, 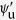 goes over *ψ*_s_. In such a case, the flow direction around *ψ*_s_ is clockwise, and the clock adjusts by delaying, as depicted in Fig. 2(c).

Special behavior occurs if *ψ*_s_ = 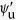, where *ψ(t*) stays at 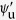 for all *t* > *t*_jetlag_; here, there is no adaptation. The corresponding jet lag in this situation is jet lag separatrix δ^*^. By solving *ψ*_s_ = 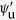, we obtain


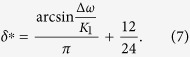


When *ω* = Ω, we have 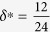 and the adaptation process is symmetric for positive and negative *δ*. For *ω* < Ω, we have 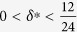. This result is intuitive in that for *ω* < Ω, it is easier for the clock system to delay because the clock system is intrinsically slower than the daily rhythm, and indeed, the clock system adapts to local time by delaying even for advance jet lags larger than δ^*^.

Hereafter, we set 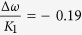, corresponding to δ^*^ ≈ +10.5/24 d, which we expect to be an appropriate value for mice. Time-series of *ψ(t*) with *K*_1_ = 1.0, Δ*ω* = −0.19 and *K*_2_ = 0 is shown in Fig. 2(d).

### Slow adaptation and desynchrony for positive time shifts

In our previous study^14^, we observed a remarkable asymmetry in the adaptation time for advance and delayed jet lag in mice. Adaptation time can be defined by PS_50_, which represents the 50% phase-shift value of the behavioral rhythm. We found that PS_50_ values were approximately five days and three days for the advance and delayed jet lag of eight hours, respectively. In addition, we reported that daily oscillations of clock gene expression became very weak for several days after advance jet lag of eight hours was experienced. Our study of SCN sections presented in ref. 14 suggests that this was due to desynchrony among clock cells rather than oscillation termination in individual cells.

Our model reproduces the above behaviors quite well. For *K*_2_ = 0, adaptation for *δ* = +8/24 is considerably slower than for *δ* = −8/24, as shown in Fig. 2(d). We also measured PS_50_ values for various *δ* values, shown in Fig. 3. We observe here that adaptation is generally slower for positive *δ* values than for negative *δ* values. In particular, there is a sharp peak around the jet lag separatrix. This very slow adaptation occurs because of the following reason. For *δ* = δ^*^, *ψ(t*) becomes 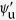 after jet lag occurs. Because *ψ(t*) = 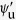 is a fixed point, i.e., 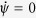, adaptation time is infinitely large. When *δ* is close to δ^*^, 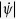 is very small just after jet lag occurs, resulting in slow adaptation.

When the AVP interaction is present but weak as compared to the VIP interaction (i.e., *K*_2_ = 0.2, *α* = 2.0), the behavior is similar to the case in which the AVP interaction is absent. Figure 4(a–c), which show the relative phases of the oscillators and the mean of the relative phases, actually resembles Fig. 2(d). Moreover, there is no irregularity in output *G(t*) of the system, as shown in Fig. 4(d).

In the presence of the AVP interaction with its strength comparable to that of the VIP interaction (i.e., *K*_2_ = 1.2, *α* = 2.0), the behavior quantitatively changes, as is evident in Fig. 4(e–g). In particular, for *δ* = +8/24 ≈ δ^*^ (i.e., Fig. 4(f)), the phase difference between oscillators 1 and 2 becomes temporally very large, i.e., desynchrony occurs. Correspondingly, the output of the system is strongly dampened for *δ* = +8/24, illustrating using a blue curve in Fig. 4(h). Moreover, PS_50_ for various *δ* values (i.e., the blue symbols in Fig. 3) shows that compared to the case of *K*_2_ = 0, the asymmetry between advance and delayed jet lag is further enhanced, and the width of the region of slow adaptation, which is present around *δ* near δ^*^, becomes much wider.

We explain the mechanism underlying the desynchrony using Fig. 5, which schematically depicts the adaptation process for small (Fig. 5(a–c)) and large (Fig. 5(d–f)) AVP interaction strengths. Note that the flow direction, depicted as red and blue curves and open arrows, are based only on the VIP interaction; the actual dynamics are determined by a combination of the VIP and AVP interactions. More specifically, for *t* < *t*_jetlag_, the two oscillators are at their stable states (i.e., the filled circles shown in Fig. 5(a,d)), at which the VIP and AVP interactions are balanced. The flow is rotated by *δ*Ω due to jet lag at *t* = *t*_jetlag_, as shown in Fig. 5(b,c,e,f), and *ψ*_1_(*t*) and *ψ*_2_(*t*) approach new stable states (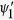, 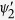, filled circles) for *t* > *t*_jetlag_ from the original stable states, represented as shaded circles.

At *t* = *t*_jetlag_, *ϕ*_0_ in equations (1b) and (1c) abruptly jumps, while the phase difference between oscillators 1 and 2 are maintained. Because the VIP interaction becomes stronger for larger *ϕ*_0_ − *ϕ*_1,2_, the VIP interaction dominates the AVP interaction for *t* > *t*_jetlag_ for a while. Figure 5(b,e) shows the case in which *δ* > 0 is sufficiently smaller than δ^*^. The initial states of oscillators 1 and 2 (i.e., shaded circles) are on the counterclockwise flow (i.e., blue curves), thus they both go counterclockwise. When *δ* is close to δ^*^, the phase difference between oscillators 1 and 2 matters, as shown in Fig. 5(c,f). In Fig. 5(c), two oscillators are on the same flow stemmed from the VIP interaction, thus they go counterclockwise together. In contrast, in Fig. 5(f), two oscillators are on opposite flows, thus they initially go in opposite directions and the phase difference increases with time; however, once the oscillators becomes very far, the effect of the AVP interaction overwhelms the VIP interaction. Because the AVP interaction tries to keep these two oscillators synchronized with some phase difference, one of the oscillators eventually changes its direction to synchronize to the other oscillator. Figure 5(f) corresponds to the case in which this occurs for oscillator 2, thus the oscillators finally re-entrain by advancing their phases, which is actually the case, as shown in Fig. 4(g). For even larger *δ* values, this occurs for oscillator 1 and the oscillators again re-entrain, this time by delaying their phases. In any case of *δ* ≈ δ^*^, the phase difference between the oscillators temporally becomes large, i.e., desynchrony occurs.

Further, desynchrony causes the adaptation of mean phase Φ slower, because the VIP interactions acting on two oscillators oppose one another during desynchrony, thus the total effect is canceled out. We can more clearly understand the effect of desynchrony on the adaptation process through the differential equation that mean phase Φ obeys. Calculations using equations (1) and (4) with the condition *R* > 0 yield (see Supplementary Information C)


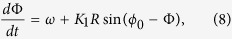


which implies that the synchronization of Φ to *ϕ*_0_ is slow when synchronization level *R* is small because the effective coupling strength appearing in this equation is proportional to *R*. As previously noted, the region of *δ* in which slow adaptation occurs (e.g., PS_50_ > 3) is much wider for *K*_2_ = 1.2 than for *K*_2_ = 0. This occurs because synchronization level *R* considerably decreases for a substantial amount of time after jet lag in this region of *δ*.

## Prediction and experimental confirmation

From our mathematical model, we found that the slow adaption process near the jet lag separatrix occurs because the system approaches an unstable state. Here, desynchrony inevitably occurs, which further disrupts the adaptation process. We can thus expect that adaptation to jet lag close to the jet lag separatrix can be accelerated if jet lag is split in half over two days, which we refer to as two-step jet lag, because in this case, the system is expected to be kept far from the unstable state during the adaptation process.

We first tested this idea in our mathematical model. The two-step jet lag of eight hours in advance 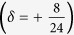 was provided as follows. The first jet lag of 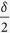 was provided at 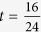, then the second jet lag of 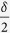 was introduced at the same time on the next day, i.e., 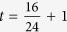. Our numerical results indeed indicated that adaptation was faster than the case of one-step jet lag, as shown in Fig. 6(a) and Fig. 4(f), respectively. We also observed that desynchrony between oscillators 1 and 2, which slows the adaptation process, is reduced for two-step jet lag, resulting in stronger oscillations in the SCN output, as shown in Fig. 6(b).

To more conveniently compare our theoretical and experimental results, we defined the onset time as the time at which the mean phase Φ passes a certain value Φ_onset_. In our model, we set Φ_onset_ = 3.0 because then the onset time is similar to that in mice, which is c.a. 12 hours. Time courses of onset time are presented in Fig. 6(c), which shows an accelerated adaptation for two-step jet lag versus that of one-step jet lag. Further, we tested our method here for various jet lags, as shown using the red symbols in Fig. 3. Two-step jet lag resulted in faster adaptation for around 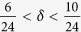 than one-step jet lag (the blue symbols), indicating the usefulness of this method for the most severe cases of jet lag; however, our method led to slower adaptation for jet lag consisting of too large an advance or delay, in which the adaptation is already relatively fast, thus we do not need any additional management.

Next, to verify the validity of the model, we measured locomotor activity of mice subjected to the one-step or two-step jet lags. Some mice were first subjected to the two-step jet lag, then to one-step jet lag, whereas other mice were subjected to one-step jet lag, then to two-step jet lag. As shown in Fig. 7, the average onset of locomotor activity under the two-step jet lag re-entrained faster as compared with that of one-step jet lag. For a quantitative comparison of the adaptation speed between one-step and two-step jet lags, we calculated PS_50_. Paired t-test analysis revealed that PS_50_ under the two-step jet lag was significantly smaller than that of one-step jet lag, as shown in Fig. 7(d) (*P* = 0.0014).

## Discussion

Our proposed mathematical model minimally incorporates the hierarchical structure in the SCN. Despite a very limited number of model variables and parameters, our model successfully reproduced several experimental results in mice. We demonstrated that our model can predict the response to jet lag and concluded that mice cope better with jet lag when they are pre-sensitized to jet lag. We believe this method would also be worth testing in humans, for example by waking up earlier by four hours on the day before a long-distance eastbound trip.

**Figure 1:**
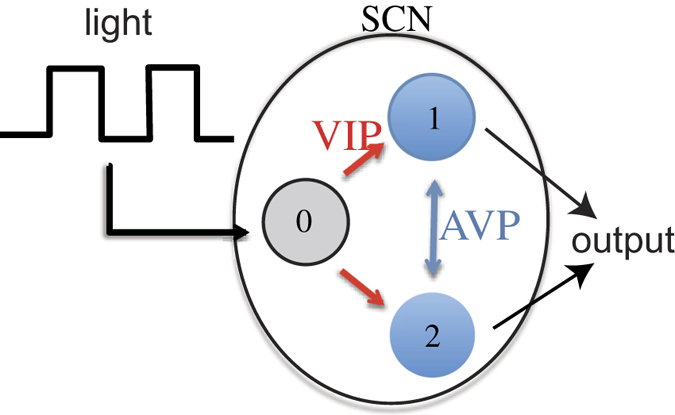
Based on the anatomical structure of the SCN, a schematic of our model consisting of three oscillators (numbered 0, 1, and 2). Oscillator 0 represents a group of neurons receiving input from the retina, whereas oscillators 1 and 2 represent groups of neurons receiving input from neurons of oscillator 0.

**Figure 2:**
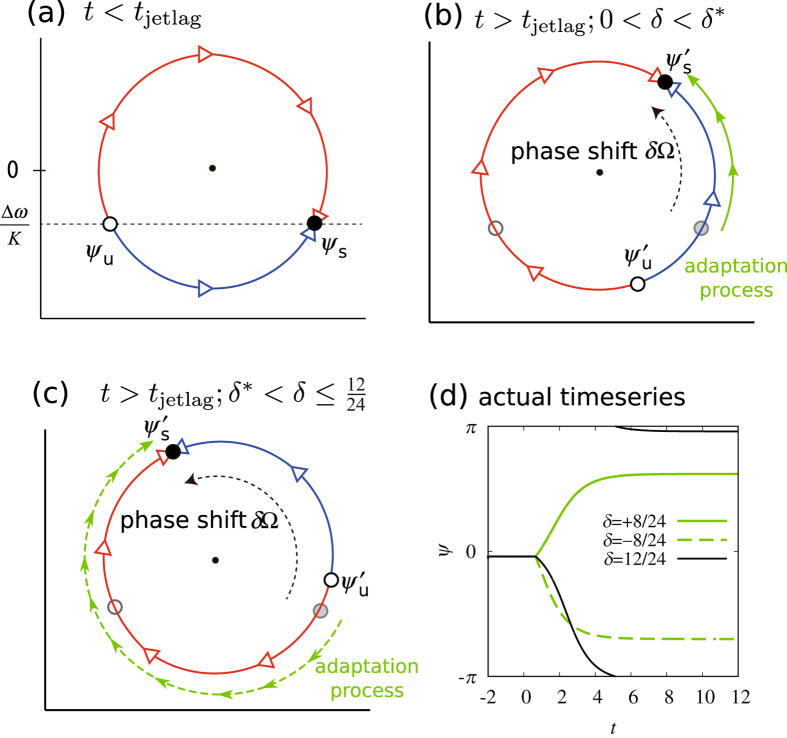
Illustrating the flow direction of relative phase *ψ(t*), depicted on a unit circle using red and blue curves and open arrows (**a**) before jet lag and after jet lag (**b**) smaller and (**c**) larger than the jet lag separatrix δ^*^. The flow direction around *ψ*_s_ (which is a stable fixed point before jet lag) is counterclockwise and clockwise in (**b**,**c**), respectively. Thus, *ψ(t*), which converges to *ψ*_s_ in (**a**), will increase or decrease to approach 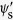 in (**b**,**c**) after jet lag, respectively. The green curve illustrates the trajectory of *ψ*. Finally, we present (**d**) the actual time series of *ψ(t*) for different values of jet lag *δ*. The clock system re-entrains by advancing its phase for *δ* < δ^*^ but by delaying it for 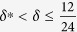, where jet lag separatrix δ^*^ is c.a. 10.5/24.

**Figure 3:**
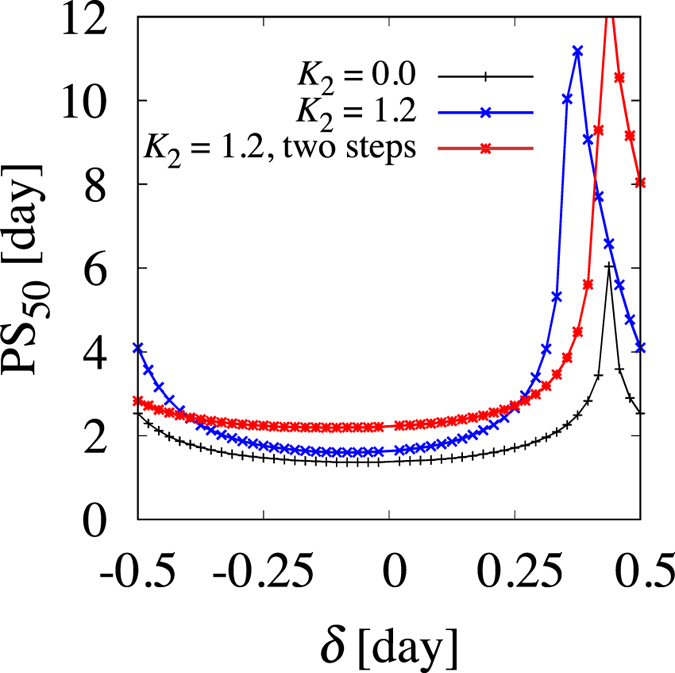
Adaptation time (PS_50_) vs various values of jet lag *δ*. PS_50_ is defined by the 50% phase-shift value of *ψ*( = *ψ*_1,2_) for *K*_2_ = 0.0 and Ψ for *K*_2_ = 1.2.

**Figure 4:**
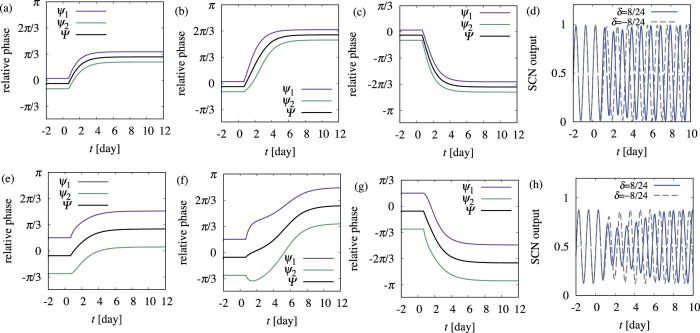
Adaptation process in the presence of (**a**–**d**) weak and (**e**–**h**) strong AVP interactions. Relative phases, defined by *ψ*_*i*_ = *ϕ*_*i*_ − Ω*t* and Ψ = Φ − Ω*t*, are plotted for (**a**,**e**) *δ* = 4/24, (**b**,**f**) *δ* = 8/24, and (**c**,**g**) *δ* = −8/24. In (**d**,**h**), SCN output *G(t*) is plotted for *δ* = ±8/24.

Jet lag separatrix is the key concept for understanding and coping with jet lag. Jet lag separatrix exists generally in oscillatory systems entrained by weak periodic stimuli. This is mathematically ensured if the system is close to a saddle-node bifurcation, which describes a transition between non-entrained to entrained states for weak forcing^35^,^36^. Near the bifurcation point, an unstable solution exists, which underlies the jet lag separatrix. For a phase oscillator, this unstable solution is depicted as a open circle in Fig. 2(a). We thus expect that the jet lag separatrix should be found in a variety of organisms ranging from single cell organisms to mammals. However, for strong periodic stimuli, there is a case in which the unstable solution does not exist^35^. In such a case, the concept of the jet lag separatrix is not meaningful, and we expect that the considerable growth of adaption time around certain *δ* would not be observed. In single cell organisms, the circadian clock should be strongly influenced by light stimuli. We thus suspect that the jet lag separatrix could disappear for the LD cycle with strong amplitude. In contrast, in our model, we hypothesized that the main part of the circadian clock (oscillators 1 and 2) are influenced via oscillator 0, as shown in Fig. 1. There, the strength *K*_1_ is assumed to be independent of the strength *K*_0_ of light stimuli, thus jet lag separatrix persists for any light intensity. We expect that this is actually the case in mammals.

**Figure 5:**
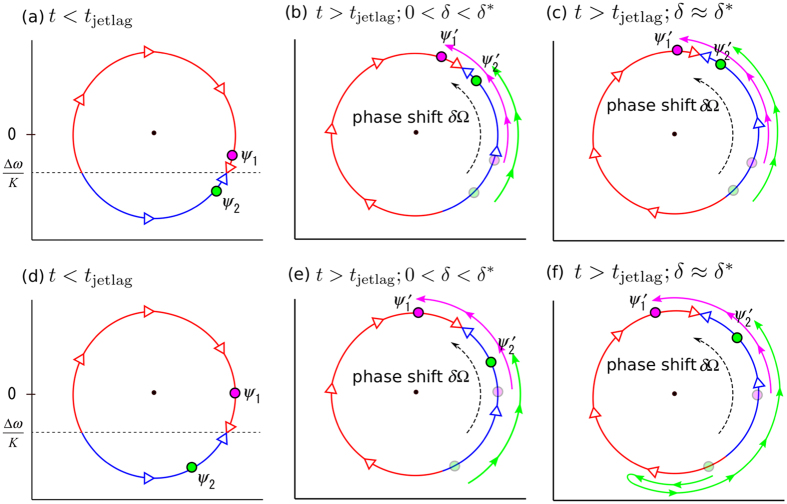
Schematic presentation of the adaptation process in the presence of (**a**–**c**) weak and (**d**–**f**) strong AVP interactions, where (**b**,**c**,**e**,**f**) correspond to (**b**,**c**,**f**,**g**) in Fig. 4, respectively. The flow direction depicted on each circle, shown as red and blue curves and open arrows, is based only on the action of the VIP coupling, i.e., the same as that of Fig. 2(d). The pink and green curves illustrate the trajectories of *ψ*_1_ and *ψ*_2_, respectively.

**Figure 6:**
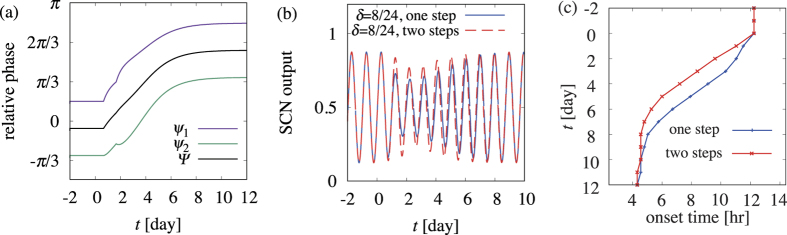
The adaptation process for two-step jet lag, showing (**a**) relative phase, (**b**) SCN output, and (**c**) onset time.

**Figure 7:**
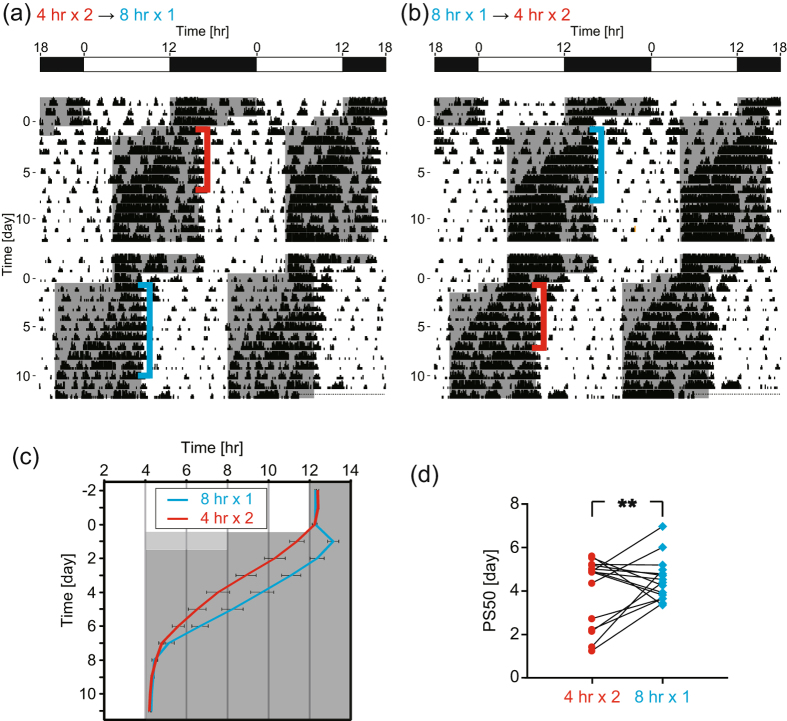
Experimental confirmation: Faster behavioral re-entrainment in the mice subjected to two-step 4-hr jet lags compared with one-step 8-hr jet lag. (**a**) Representative double-plotted actogram of mice subjected to 4-hr jet lag in LD cycle twice in the consecutive days. After 3 weeks, they were subjected to one-step 8-hr jet lag. (**b**) Representative double-plotted actogram of mice subjected to one-step 8-hr jet lag in LD cycle. After 3 weeks, they were subjected to 4-hr jet lag in LD cycle twice in the consecutive days. (**c**) Average onset of locomotor activity under one- and two-step jet lag (mean ± s.e.m.; *n* = 15 each). (**d**) Pairwise comparison in PS_50_ values (^**^P = 0.0014, paired t-test, *n* = 15).

We comment on the difference between our present model and previously proposed models. In our present model, each oscillator describes a representative oscillator in a subpopulation of cells in the SCN. One of the authors previously proposed a model in which each subpopulation is modeled by a large population of heterogeneous oscillators^24^. The same author also proposed a model similar to the present model^25^. All of these models including our original model^14^ reproduced experimental findings^14^. In particular, long-lasting desynchrony was observed for a certain range of jet lags near the jet lag separatrix. The merit of our present model over these previously proposed models is its simplicity while keeping the essential assumption of the hierarchical organization in the SCN. A further simplified model, in which a large number of oscillators are coupled globally, was proposed very recently by another group^26^. This model has only one group, i.e., no hierarchical structure, and the system behaves as a single oscillator. There, long-lasting desynchrony was not reported. The long-lasting desynchrony observed in our models is attributed to the conflict between the VIP and AVP influences, as schematically shown in Fig. 5(f). The hierarchical organization plays an vital role in this.

As noted above, it is well known that workers in rotating shifts (i.e., rotators) have more sleep-wake cycle disruption and are therefore at an increased risk of various diseases^1^,^2^,^3^,^4^,^5^,^6^,^7^,^8^. Based on our results, we suspect that the bad timing of phase shift of one’s daily rhythm desynchronizes the clock cells in the SCN of rotators, which may be one of the main causes of reported health problems. We feel it is of great interest to use our model to predict the effect of shift work on the circadian clock. Such an application will make more sense if we fit the parameter values of our model to human data, such as melatonin density in the blood. In particular, we are curious as to how the resulting value of jet lag separatrix will determine the response of the clock system to substantial jet lags. Considering the circadian period of humans in constant darkness is longer than that of mice, the jet lag separatrix might also be closer to 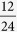. Possibly, the morning person or night owl have smaller and larger values of jet lag separatrix, respectively. It should be helpful to predict the effect of shift work and long-distance trips using our model with parameter values fit to each individual.

Finally, we note the relevance of the singularity of circadian rhythm, a well-known mathematical concept proposed by A. Winfree^17^, to our study. Winfree pointed out that a perturbation of a particular strength to a limit-cycle oscillator at a particular phase leads the system to a singular point in the state space, thus resulting in oscillation arrest. Moreover, he experimentally confirmed this for the circadian clock of fruit flies. For this phenomenon to occur, one generally needs to precisely tune two parameters of perturbation, e.g., phase and strength. Although in our case we have only one parameter (i.e., the amount of jet lag), the oscillation amplitude considerably dampens for a substantial amount of time after jet lag occurs, which is seemingly similar to oscillation arrest; however, our interpretation is that this is not because the system approaches a singular point but because SCN cells split into two groups, in one of which cells advance the phases of their cellular clocks and in the other of which cells delay the phases. As we have shown with our mathematical model, the latter may occur for a range of *δ* without careful tuning of other parameters, which is therefore expected to be relevant to our daily lives.

## Methods

All animal care and experiments conformed to the Guidelines for Animal Experiments of Kyoto University, and were approved by the Animal Research Committee of Kyoto University. Caged wild-type C57BL/6 mice, all eight-week-old male, were housed individually in light-tight ventilated closets within a temperature- and humidity-controlled facility with ad libitum access to food and water. The animals were entrained on a 12-hour-light (~200 lux fluorescent light) and 12-hour-dark cycle for at least two weeks to synchronize (i.e., entrain) their circadian clocks to the ambient light-dark cycle. Next, the mice where subjected to LD cycles that were phase-advanced by eight hours or four hours twice over consecutive days. Locomotor activity was recorded in five-minute bins with a passive (pyroelectric) infrared sensor (FA-05 F5B; Omron), and the data obtained were analyzed using Clocklab software (Actimetrics) developed on MATLAB (Mathworks). PS_50_ values were determined as previously described^14^.

## Additional Information

**How to cite this article:** Kori, H. *et al*. Accelerating recovery from jet lag: prediction from a multi-oscillator model and its experimental confirmation in model animals. *Sci. Rep.* **7**, 46702; doi: 10.1038/srep46702 (2017).

**Publisher's note:** Springer Nature remains neutral with regard to jurisdictional claims in published maps and institutional affiliations.

## Supplementary Material

Supplementary Information
