# Supplementary material for: Accelerating recovery from jet lag: prediction from a multi-oscillator model and its experimental confirmation in model animals
Source: Sci Rep. 2017 Apr 26;7:46702. doi: 10.1038/srep46702 (PMC5405409; doi:10.1038/srep46702)
Supplement: Supplementary Information [file srep46702-s1.pdf]

Supporting Information for
”Accelerating Recovery from Jet Lag: Prediction From a Multi-oscillator Model and Its Experimental Confirmation in Model Animals”

H. Kori,[1] Y. Yamaguchi[2] and H. Okamura[2]
[1]Department of Information Sciences, Ochanomizu University, Tokyo 112-8610, Japan.
[2]Department of Systems Biology, Graduate School of Pharmaceutical Sciences, Kyoto University, Kyoto 606-8501, Japan.

A Jet lag separatrix in mice

Figure S1 displays representative locomotor activity in mice. When the mice were subjected to phase advance of the LD cycle by eight hours, almost all re-entrained by advancing their daily rhythm of locomotor activity, i.e., by waking up earlier every day to adjust their daily rhythm to the shifted LD cycle. In contrast, when the mice were subjected to phase shift of the LD cycle by 12 hours, all re-entrained by delaying their daily rhythm of locomotor activity. These results indicate that the jet lag separatrix exists between the advancement of eight hours and 12 hours, possibly around the advancement of 10 hours.

B Derivation of Eq. (5)

A rough estimation to the synchronized state in Eqs. (1b) and (1c) can be obtained by linearizing the coupling terms, yielding

$$\dot{\phi}_1 = \omega + K_1(\phi_0 - \phi_1) + K_2(\phi_2 + \alpha - \phi_1), \tag{S1a}$$

$$\dot{\phi}_2 = \omega + K_1(\phi_0 - \phi_2) + K_2(\phi_1 - (\phi_2 + \alpha)). \tag{S1b}$$

By solving $\dot{\phi}_1 = \dot{\phi}_2$, we obtain Eq. (5).

C Derivation of Eq. (8)

We define synchronization level $R \geq 0$ and collective phase $\Phi(t)$ as the norm and phase of the Kuramoto complex order parameter, respectively, i.e.,

$$Re^{\mathrm{i}\Phi} = \frac{1}{2}(e^{\mathrm{i}\phi_1} + e^{\mathrm{i}\phi_2}). \tag{S2}$$

From Eq. (S2), it follows that

$$R = \frac{1}{2}\sqrt{(\cos\phi_1 + \cos\phi_2)^2 + (\sin\phi_1 + \sin\phi_2)^2} \tag{S3}$$

$$= \left|\cos\frac{\Delta\phi}{2}\right|, \tag{S4}$$

where $\Delta\phi = \phi_1 - \phi_2$. It also follows that

$$\cos\Phi = \frac{\cos\frac{\phi_1+\phi_2}{2}\cos\frac{\Delta\phi}{2}}{\left|\cos\frac{\Delta\phi}{2}\right|}, \tag{S5}$$

$$\sin\Phi = \frac{\sin\frac{\phi_1+\phi_2}{2}\cos\frac{\Delta\phi}{2}}{\left|\cos\frac{\Delta\phi}{2}\right|}. \tag{S6}$$

Equations (S5) and (S6) imply that

$$\Phi = \begin{cases} \frac{\phi_1+\phi_2}{2} & \text{if } \cos\frac{\Delta\phi}{2} \geq 0, \\ \frac{\phi_1+\phi_2}{2} + \pi & \text{otherwise.} \end{cases} \tag{S7}$$

We thus have

$$\phi_1 = \begin{cases} \Phi + \frac{\Delta\phi}{2} & \text{if } \cos\frac{\Delta\phi}{2} \geq 0, \\ \Phi + \frac{\Delta\phi}{2} + \pi & \text{otherwise,} \end{cases} \tag{S8}$$

and

$$\phi_2 = \begin{cases} \Phi - \frac{\Delta\phi}{2} & \text{if } \cos\frac{\Delta\phi}{2} \geq 0, \\ \Phi - \frac{\Delta\phi}{2} + \pi & \text{otherwise}, \end{cases} \tag{S9}$$

By taking the time-derivative of Eq. (S2), we obtain

$$\dot{R} + \mathrm{i}R\dot{\Phi} = \frac{\mathrm{i}}{2}\left(\dot{\phi}_1 e^{\mathrm{i}(\phi_1-\Phi)} + \dot{\phi}_2 e^{\mathrm{i}(\phi_2-\Phi)}.\right) \tag{S10}$$

By assuming $R > 0$ and using Eq. (S7), we obtain

$$\dot{\Phi} = \frac{1}{2}\left(\dot{\phi}_1 \frac{\cos(\phi_1 - \Phi)}{\left|\cos\frac{\Delta\phi}{2}\right|} + \dot{\phi}_2 \frac{\cos(\phi_2 - \Phi)}{\left|\cos\frac{\Delta\phi}{2}\right|}\right) \tag{S11}$$

$$= \frac{1}{2}(\dot{\phi}_1 + \dot{\phi}_2). \tag{S12}$$

Substituting Eqs. (1b) and (1c) into Eq. (S12) and using Eqs. (S4) and (S7), we obtain

$$\frac{d\Phi}{dt} = \omega + \frac{K_1}{2}\{\sin(\phi_0 - \phi_1) + \sin(\phi_0 - \phi_2)\} \tag{S13}$$

$$= \omega + K_1 \cos\frac{\Delta\phi}{2} \sin\left(\phi_0 - \frac{\phi_1 + \phi_2}{2}\right) \tag{S14}$$

$$= \omega + K_1 \left|\cos\frac{\Delta\phi}{2}\right| \sin(\phi_0 - \Phi) \tag{S15}$$

$$= \omega + K_1 R \sin(\phi_0 - \Phi), \tag{S16}$$

which is Eq. (8).

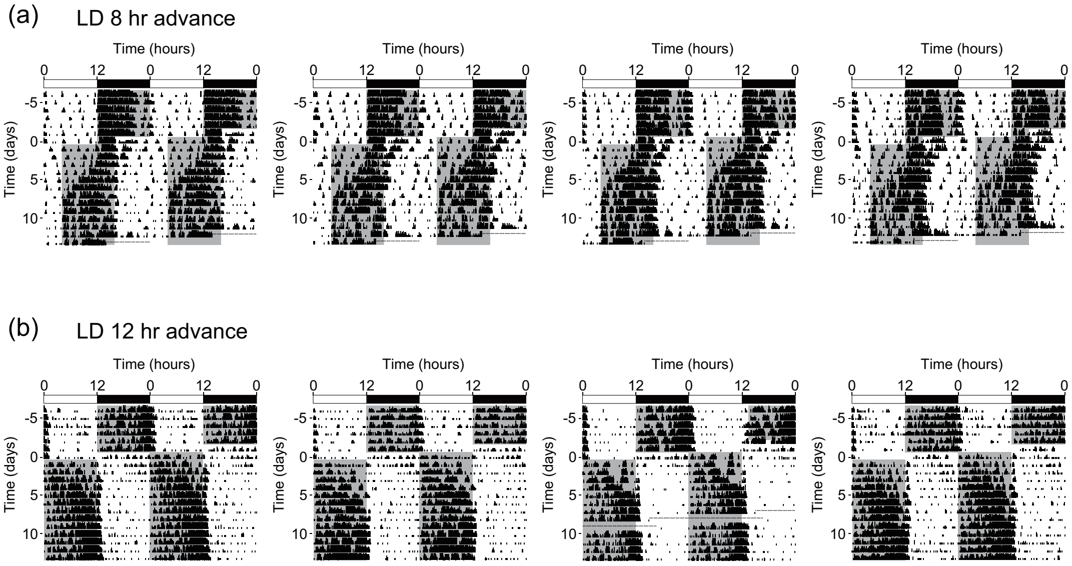

Figure S1. Locomotor activity of mice, i.e., representative double-plotted actograms of mice subjected to (a) eight-hour jet lag and (b) 12-hour jet lag.
